# Examining interhemispheric processing and task demand in lexical decision-making: insights from lateralized visual field paradigm

**DOI:** 10.3389/fpsyg.2023.1208786

**Published:** 2023-06-15

**Authors:** Sangyub Kim, Kichun Nam

**Affiliations:** ^1^Wisdom Science Center, Korea University, Seoul, South Korea; ^2^School of Psychology, Korea University, Seoul, South Korea

**Keywords:** signal detection theory, laterality, interhemispheric interaction, visual word recognition, response bias

## Abstract

This study aimed to investigate the influence of task demand on the uni−/bi-hemispheric processing of lexical decision-making. Two types of nonwords were used in parafoveal and foveal lexical decision tasks (LDTs) to manipulate task demand. In Experiment 1, a visual half-field paradigm was utilized to evaluate the unihemispheric strategy in lexical decision, which revealed a significant response bias toward “word” at the RVF/LH in the pseudoword LDT in contrast with the nonword LDT, indicating the strategic use of orthographical legality in LH for word-pseudoword lexical decision. In Experiment 2, the study evaluated whether foveal lexical decision follows the orthographical legality strategy of LH in pseudoword LDT relative to the nonword LDT. The results showed a response bias toward “word” in the foveal pseudoword LDT in contrast with the foveal nonword LDT, suggesting the recruitment of LH in foveal pseudoword LDT. These findings support the left-dominant bihemispheric processing in foveal lexical decision and contribute to our understanding of the mechanisms underlying lexical decision-making.

.

## 1. Introduction

The interhemispheric processing of visual words during lexical decision tasks has been a longstanding area of investigation, with a particular focus on the mechanisms by which the left hemisphere (LH) and right hemisphere (RH) interact. Previous studies have demonstrated that the interplay between the two hemispheres plays a critical role in lexical decision making in languages (e.g., Ibrahim and Eviatar, 2012; Kim et al., 2020). Despite the widely accepted specialization of LH for language processing, it is posited that both hemispheres continuously collaborate to establish lexical criteria throughout the processing flow involved in lexical decision. Nonetheless, the precise manner in which LH and RH interhemispherically establish criteria for word judgments in lexical decision tasks remains unresolved.

### 1.1. Task demand in lexical decision task

Task demand, called task difficulty, is a crucial factor that affects interhemispheric interaction, as highlighted in previous studies (Bradshaw et al., 1979; Chiarello et al., 1988). Task demand refers to the cognitive requirements that are necessary to perform a task, which can have an impact on the subject’s ability to complete the task successfully. An example of such a task is the lexical decision task, which manipulates the difficulty of the task by varying the orthographic legality of nonwords. Specifically, participants are expected to experience greater difficulty when making a lexical decision between a word and an orthographically legal nonword, such as a pseudohomophone (e.g., brain [word]–brane [pseudoword]), compared to a word and an orthographically illegal nonword (e.g., brain [word]–bqwkz [nonword]). Thus, the task demand significantly affects the subject’s criterion for judging a word during the lexical decision, which, in turn, influences the establishment of response bias for lexical decision. A high criterion for a word results in a conservative decision, while a low criterion leads to a liberal decision. The differences in the decision criterion between the two hemispheres may lead to a distinct pattern of interhemispheric interaction.

### 1.2. Examination of hemispheric processing through visual half-field study

To investigate the hemispheric processing in lexical decision, we employed the visual half-field paradigm that presents arbitrary letter strings in either the left visual field (LVF) or the right visual field (RVF), projecting to the contralateral hemisphere. For example, the LVF presentation propagates the letter strings to the RH and vice versa. This experimental manipulation allows for the study of both hemispheric lateralization and interhemispheric communication by presenting the letter strings at the parafoveal vision. Our study aims to contribute to the existing literature on this topic by examining the impact of task demand on hemispheric interactions during lexical decision. Previous research has shown that presenting visual words simultaneously in both visual fields (bilateral visual field, BVF) results in better performance than presenting the word in only one visual field (unilateral visual field, UVF; e.g., Mohr et al., 2007; Kim et al., 2022). This suggests that LH and RH work together to recognize words, even though they have asymmetric efficiency in visual word processing. By analyzing the performance of participants under different task demands, we aim to gain a better understanding of the specialized functions of LH and RH and their strategic interaction to overcome each other’s weaknesses.

### 1.3. Manipulation of task demand using nonword type for hemispheric processing

The current study aims to investigate the interhemispheric interaction of LH and RH through parafoveal and foveal lexical decision tasks by manipulating task demand using two types of nonwords. Prior research has predominantly employed pseudoword stimuli to examine interhemispheric interactions during lexical decision-making (e.g., Chiarello et al., 1988), reporting a bias toward “word” responses in the RVF/LH and “nonword” responses in the LVF/RH. These findings suggest that the RVF/LH may utilize an orthographic legality strategy to accept permissible letter sequences as words. However, these conclusions remain uncertain as prior research did not incorporate orthographically illegal nonwords in their experiment. The present study overcomes this limitation by concurrently presenting pseudowords and nonwords, allowing for a focused analysis of the impact of nonword type on unihemispheric lexical decision-making and elucidating the tactical differences in lexical decision-making between LH and RH, as well as their interplay.

### 1.4. Signal detection measures for hemispheric processing

Previous studies consistently demonstrate a right visual field advantage (RVFA) for letter string processing, where a “yes (word)” response tendency is observed for stimuli presented in the RVF/LH, and a “no (nonword)” response bias is observed for those presented in the LVF/RH (Chiarello et al., 1984, 1986, 1988; Babkoff et al., 1985). These findings suggest distinct processing strategies across the hemispheres, with LH relying on orthographic legality for lexical decision making, and RH potentially relying more heavily on other visual-perceptual attributes or visual familiarity, or requiring greater evidence to make a lexical decision due to a lack of useful lexical information (Hellige and Webster, 1979; Warrington and James, 1986). To further examine the differential processing strategies across the hemispheres, we utilized signal detection measures, sensitivity (d’) and response bias (β), which are independent indicators from each other (McNichol, 1972; Willemin et al., 2016; Huang and Ferreira, 2020; Batailler et al., 2022). Sensitivity reflects the ability to discriminate between signal and noise, while response bias reflects a predisposition toward a particular response. These two measures provide additional implications beyond simply measuring response times and accuracy. Sensitivity allows for the assessment of discrimination between stimuli types, such as words and nonwords, offering insight into the differential processing strategies according to their type. Response bias can evaluate the distinctive strategy according to task demands/difficulty, for example, in high task difficulty, the responses are expected to be more conservative by showing bias toward specific responses, providing insight into scrutinizing the differential strategy for responses. Thus, the current study aims to examine the uni−/bi-hemispheric processing of nonwords in lexical decision making and the impact of task difficulty on the differential strategy employed by the hemispheres. We hypothesize that changes in task difficulty will result in differential processing strategies across the hemispheres for nonwords in lexical decision making.

### 1.5. Theoretical models of hemispheric processing in lexical decision

In the realm of hemispheric processing for lexical decision-making, three theoretical models have been proposed: parallel processing, inhibition, and cooperation. Parallel processing models, as put forth by Raab (1962), Miller (1982), and Allen (1983), posit that both hemispheres are engaged in interhemispheric processing but function independently without interacting with each other. The modalities of each hemisphere are processed separately, with no interaction between the hemispheres to attain the response criterion (Miller, 1982). The assumption is that parallel processing between the hemispheres occurs if there are no differences in foveal and parafoveal recognition performances, as measured by signal detection measures such as sensitivity and response bias, as well as behavioral responses such as response times and accuracy. On the other hand, inhibition models, proposed by Mackavey et al. (1975), Boles (1979, 1990, 1995), and Allen (1983), suggest that inhibition or suppression occurs between the hemispheres in bilateral presentation when both hemispheres have the capability to perform a particular task. Two hypotheses have been proposed in previous studies: the mutual inhibition hypothesis (Mackavey et al., 1975; Boles, 1979) and the homologous activation hypothesis (Boles, 1990, 1995), both of which suggest that activation of one hemisphere suppresses the other hemisphere in bilateral presentation. Inhibitory interaction leads to a decrease in sensitivity, slower response times, and/or less accurate responses in lexical decision. In contrast, cooperation models propose that hemispheric processing is augmented when both hemispheres cooperate after bilateral presentation. The processing gain is the most significant difference from other hemispheric models. Allen (1983) has explained the positive gain in bilateral presentation with two possibilities. Firstly, the two hemispheres complement each other’s weaknesses through cooperative interaction. Secondly, both hemispheres are involved in nearly the same process, resulting in interactive benefits in overall performance. Cooperative interaction between the hemispheres leads to greater sensitivity, faster response times, and/or more accurate responses in lexical decision. Given LH’s superiority in language processing, RH’s involvement in word processing is expected to determine the mode of interhemispheric processing in lexical decision.

### 1.6. The current study

Therefore, we hypothesized that the pseudoword LDT would primarily activate LH due to its high task difficulty, while the nonword LDT would engage both LH and RH due to its relatively low difficulty level. To test this hypothesis, we focused on the response bias in signal detection measures and conducted two LDT experiments that manipulated the type of nonwords. In the first experiment, we employed the visual half-field presentation paradigm to examine the hemispheric locus of response bias induced by nonword type. Specifically, we expected that participants would exhibit a higher response bias in the RVF/LH than in the LVF/RH during parafoveal pseudoword LDT compared to parafoveal nonword LDT, reflecting LH’s reliance on orthographic legality. In the second experiment, stimuli were presented at the foveal vision to determine which hemisphere’s strategy was primarily utilized and how interhemispheric interaction manifests during foveal word recognition. If foveal lexical decision mainly follows a left-centered strategy with orthographic legality, we expected to observe a higher response bias in the foveal pseudoword LDT than in the foveal nonword LDT. Alternatively, we anticipated observing a non-significant response bias in both foveal pseudoword and nonword LDTs if the task predominantly engages both hemispheres.

## 2. Experiment 1

In this study, Experiment 1 aimed to investigate the unihemispheric strategy in lexical decision making, with a focus on the influence of nonword type on response bias in the visual half-field presentation paradigm. Drawing on previous research (Chiarello et al., 1984, 1986, 1988; Babkoff et al., 1985), we hypothesized that parafoveal pseudoword lexical decision tasks would elicit a higher response bias toward “word” in the RVF/LH compared to the LVF/RH, as opposed to parafoveal nonword LDT. This response pattern would ensure the engagement of the orthographic legality strategy in LH, while RH may rely on different strategies such as visual-perceptual attributes or visual familiarity, as previously proposed (Hellige and Webster, 1979; Warrington and James, 1986). With this study, we aim to identify the response bias locus and uncover the mechanisms of the interhemispheric interaction that underlie hemispheric asymmetry in lexical decision making, building on prior literature.

### 2.1. Methods

#### 2.1.1. Participants

In Experiment 1, two distinct experiments were conducted to examine nonword LDT (Experiment 1–1) and pseudoword LDT (Experiment 1–2), respectively. Sixty five individuals participated in Experiment 1–1, with four participants excluded due to noncompliance with the experimental protocol. Consequently, data from 61 participants (male: 29, female: 31) were included in the final analysis. The mean age of the participants in Experiment 1–1 was 23.80 years (SD: 2.34). All participants demonstrated strong right-handedness as determined by the Edinburgh handedness inventory (M: 8.05, SD: 1.83). Additionally, 56 participants completed Experiment 1–2, with two participants excluded due to noncompliance with the experimental protocol. Therefore, data from 54 participants (male: 25, female: 29) were analyzed. The mean age of the participants in Experiment 1–2 was 24.52 years (SD: 2.49), and all demonstrated strong right-handedness (M: 7.48, SD: 2.15). There was no overlap in participants between Experiment 1–1 and Experiment 1–2.

All participants in this study possessed normal or corrected-to-normal vision of both eyes and had no medical history of neurological impairments. Ethical standards established in the 1964 Declaration of Helsinki were adhered to throughout the study. This study received approval from the ethics committee of Korea University, South Korea, where the study was conducted. Prior to participation, all participants received a thorough explanation of the study’s ethics and provided informed consent. A small amount of compensation was provided for their participation. Participants were excluded from the study if they met any of the following criteria: (1) a known history of neurological impairment due to brain damage or stroke, (2) a known history of sensory organ impairments, (3) diagnosis of mental illness, or (4) a known history of substance abuse or addiction.

#### 2.1.2. Experimental task and procedure

In Experiment 1–1 and Experiment 1–2, a lateralized lexical decision task was administered to the participants using different types of nonwords. Experiment 1–1 employed orthographically illegal letter sequences, while Experiment 1–2 used orthographically legal letter sequences in the form of pseudowords. The procedures of both experiments were identical. A fixation point was presented at the center of the screen for 2,000 ms followed by the presentation of arbitrary letter strings in either the LVF or the RVF for 180 ms. After the letter strings disappeared, participants were required to determine within 2,000 ms whether the presented stimuli constituted a word or not. The letter strings were displayed horizontally within a visual angle range of 1.5° vertical and 2° (innermost) to 4.4° (outermost) horizontal.

#### 2.1.3. Apparatus

In accordance with standard practice, all participants were required to maintain a consistent distance from the monitor, resting their chin on a chinrest at a distance of 65 cm. Stimuli were presented *via* a high-quality LG monitor capable of displaying RGB colors. Participants responded to the stimuli using a keyboard positioned in front of the monitor, pressing the “/(slash)” key with the index finger of their right hand for a word, and the “z” key with the index finger of their left hand for a pseudoword. Response keys were counterbalanced across participants in each experiment. The stimuli themselves were presented in white letters on a black background, and the experiment was run using the widely utilized Experimental Psychology Software, E-prime.

#### 2.1.4. Stimuli

The present study employed 300 Korean visual words in both Experiment 1–1 and Experiment 1–2, but with different types of nonword stimuli. Specifically, Experiment 1–1 utilized 300 orthographically illegal nonwords, while Experiment 1–2 employed 300 orthographically legal pseudowords. The stimuli set of words was selected from various sources, including movies (10%), newspapers (20%), books (30%), and internet blogs or posts (40%), with reference to the Korean Sejong Corpus (Kang and Kim, 2009). Pseudowords were generated by randomly combining syllables from Korean words, whereas nonwords were created by substituting an undefined syllable in the Korean Sejong National Corpus with an arbitrary syllable [e.g., the nonword “**챹화는**” was created by replacing the syllable “**단**” in the word “**담화는**(conversation)” with the syllable “**챹**”]. Notably, the pseudowords were orthographically legal and pronounceable, whereas the nonwords were orthographically illegal but still phonologically plausible. Table 1 described the lexical variables of words, nonwords, and pseudowords employed in the current study.

**Table 1:** Lexical variables of words, nonwords, and pseudowords utilized in the present investigation.

	Length variable				Frequency variable	Semantic variable
	# of strokes	# of phonemes	# of syllables	# of morphemes	Frequency	# of objective meanings
Words	18.833 (4.532)	8.063 (1.554)	3.203 (0.532)	2.098 (0.314)	383 (959)	1.500 (1.228)
Nonwords	21.650 (4.387)	8.500 (1.496)	3.203 (0.532)	–	–	–
Pseudowords	19.647 (4.885)	8.187 (1.508)	3.203 (0.532)	–	–	–

A Latin square design was employed to ensure the counterbalancing of stimuli across two visual fields (LVF, RVF) in both Experiment 1–1 and Experiment 1–2. The stimuli set included 300 Korean words and 300 nonwords in Experiment 1–1, and 300 Korean words and 300 pseudowords in Experiment 1–2. To achieve counterbalancing, the stimuli were divided into two lists, with each list containing an equal number of stimuli from both word and nonword categories. Each list was then presented in both visual fields, ensuring that no stimulus was presented twice in the same visual field. Specifically, the stimuli presented in the LVF in list 1 of Experiment 1–1 were presented in the RVF in list 2 of Experiment 1–1, and vice versa.

#### 2.1.5. Signal detection theory

In line with signal detection theory, a yes/no decision in signal detection is influenced by two key factors. The first factor, sensitivity, refers to the degree of overlap between the signal and noise distribution (Stanislaw and Todorov, 1999). This factor provides insight into how well the observer detected the signal. An increase in sensitivity in signal detection indicates an enhanced ability to differentiate between the signal and noise. The formula for sensitivity in a yes/no task is as follows (Snodgrass and Corwin, 1988):


(1) $$A^{\prime }=.5+\left[sign\left(H-F\right)\frac{{\left(H-F\right)}^{2}+|H-F|}{4\max\left(H,F\right)-4HF}\right],$$

where H denotes the hit rate and F represents the false alarm rate. Hit rate is the likelihood of responding “yes” to signal trials, while false alarm rate indicates the likelihood of responding “yes” to noise trials. In the lexical decision task, a “hit” is recorded when the participant responds “yes” to a word trial, while a “false alarm” is recorded when the participant responds “yes” to a nonword trial. The term sign(H−F) takes a value of +1 when H−F > 0, a value of 0 when H=F , and a value of −1 when H−F < 0. Furthermore, max(H,F) denotes the maximum value between H and F.

Response bias measures the general tendency of the observer’s response toward “yes” or “no”. The magnitude of the response tendency is determined by the absolute value, with positive and negative values indicating “yes” and “no” response biases, respectively. The strength of the bias increases as the value becomes larger positively or negatively. The response bias provides information on the criteria used by the observer to make a decision in a yes/no task, which is usually based on the observer’s experience or inherent attributes related to the decision. In the case of lexical decision, for instance, observers use their lexical knowledge or experience to determine whether an arbitrary letter string is a lexical item or not. The formula for response bias in a yes/no task is given as follows (Snodgrass and Corwin, 1988):


(2) $$B^{\prime \prime }=sign\left(H-F\right)\frac{H\left(1-H\right)-F\left(1-F\right)}{H\left(1-H\right)+F\left(1-F\right)},$$

where H means hit rate and F indicates false alarm rate. And, sign(H−F) equals +1 in case of H−F > 0, equals 0 if H=F , and equals −1 if H−F < 0.

In this study, we specifically focused on the latter indicator, utilizing the formula outlined above and considering the hemispheric contribution to response bias.

#### 2.1.6. Statistical analyses

The present study aimed to investigate the effects of various factors on behavioral responses and signal detection measures in lexical decision tasks. Specifically, Experiment 1 employed a 1-between (treatment: nonword LDT vs. pseudoword LDT) and 2-within (lexicality: word vs. nonword; visual field: RVF vs. LVF) mixed ANOVA design on behavioral responses (RTs and Acc). The treatment factor represented the lexical decision performances in Experiment 1–1 (parafoveal nonword LDT) and Experiment 1–2 (parafoveal pseudoword LDT), which provided two levels of nonword LDT and pseudoword LDT. Lexicality factor distinguished words and nonwords in the tasks, showing two levels of word and nonword. Lastly, visual field factor denoted the left and right visual field that the stimuli were presented, representing two levels of RVF vs. LVF. Additionally, we performed 1-between (treatment: nonword LDT vs. pseudoword LDT) 1-within (visual field: RVF vs. LVF) mixed effect ANOVA on signal detection measures (sensitivity and response bias). The treatment and visual field factors were consistent with the analyses for the behavioral responses.

### 2.2. Results

#### 2.2.1. Behavioral responses (response times and accuracy)

The behavioral response outcomes in Experiment 1 are presented in Table 2. Initially, we conducted mixed ANOVAs with a 1-between factor (treatment: nonword LDT vs. pseudoword LDT) and 2-within factors (lexicality: word vs. nonword; visual field: RVF vs. LVF) on response times in Experiment 1. The results indicated a lack of a significant three-way interaction effect between the factors and two-way interaction effect between treatment and visual field [*F*(1, 598) = 405.037, *p* = 0.597, $\eta_{p}^{2}$ =0.000 for treatment × lexicality × visual field; *F*(1, 598) = 2.262, *p* = 0.133, $\eta_{p}^{2}$ =0.004 for treatment × visual field]. However, there were statistically significant two-way interaction effects between treatment and lexicality, and between lexicality and visual field [*F*(1, 598) = 75.579, *p* < 0.001, $\eta_{p}^{2}$ =0.112 for treatment × lexicality; *F*(1, 598) = 35.631, *p* < 0.001, $\eta_{p}^{2}$ =0.056 for lexicality × visual field]. Moreover, all of the main effects were significant [*F*(1, 598) = 136.023, *p* < 0.001, $\eta_{p}^{2}$ =0.185 for treatment; *F*(1, 598) = 201.470, *p* < 0.001, $\eta_{p}^{2}$ =0.252 for lexicality; *F*(1, 598) = 293.680, *p* < 0.001, $\eta_{p}^{2}$ =0.329 for visual field]. Subsequently, we conducted simple main effect analyses on the two-way interaction effects. The results revealed significantly faster response times in the nonword LDT than in the pseudoword LDT for words and nonwords [*F*(1, 1,198) = 7.961, *p* = 0.005, $\eta_{p}^{2}$ =0.007 for words; *F*(1, 1,198) = 320.097, *p* < 0.001, $\eta_{p}^{2}$ =0.211 for nonwords] in the treatment by lexicality interaction. Furthermore, the lexicality by visual field interaction effect indicated significantly faster response times in the RVF than in the LVF for both words and nonwords [*F*(1, 599) = 267.678, *p* < 0.001, $\eta_{p}^{2}$ =0.309 for words; *F*(1, 599) = 58.996, *p* < 0.001, $\eta_{p}^{2}$ =0.090 for nonwords]. The main effect of visual field indicated faster response times in the RVF than in the LVF. Additionally, the lexicality main effect revealed significantly faster response times for words than nonwords, and the main effect of the treatment indicates faster responses in the nonword LDT than the pseudoword LDT. These results suggest that the factors of treatment, lexicality, and visual field significantly influence response times in lexical decision tasks.

**Table 2:** Results of the response times and the accuracy at the parafoveal presentation (RVF; right visual field, LVF; left visual field) in Experiment 1–1 (nonword) and Experiment 1–2 (pseudoword).

		Experiment 1–1 (nonword)		Experiment 1–2 (pseudoword)	
		Visual field		Visual field	
		RVF	LVF	RVF	LVF
Word	RTs	619 (51)	658 (57)	633 (68)	665 (68)
	ACC	0.941 (0.058)	0.917 (0.082)	0.923 (0.100)	0.904 (0.111)
Nonword	RTs	647 (54)	666 (55)	716 (75)	731 (72)
	ACC	0.940 (0.077)	0.933 (0.089)	0.889 (0.129)	0.893 (0.124)

The values within brackets denote standard deviation.

Subsequently, we conducted 1-between (treatment: nonword LDT vs. pseudoword LDT) 2-within (lexicality: word vs. nonword; visual field: RVF vs. LVF) mixed ANOVAs on the accuracy rates. Our results revealed no significant three-way interaction effect between factors and two-way interaction effect between treatment and visual field [*F*(1, 598) = 0.635, *p* = 0.426, $\eta_{p}^{2}$ =0.001 for treatment × lexicality × visual field; *F*(1, 598) = 3.232, *p* = 0.073, $\eta_{p}^{2}$ =0.005 for treatment × visual field]. However, we found significant two-way interaction effects between treatment and lexicality, and between lexicality and visual field [*F*(1, 598) = 8.696, *p* = 0.003, $\eta_{p}^{2}$ =0.014 for treatment × lexicality; *F*(1, 598) = 22.458, *p* < 0.001, $\eta_{p}^{2}$ =0.036 for lexicality × visual field]. Additionally, our analysis showed significant main effects of treatment and visual field [*F*(1, 598) = 33.021, *p* < 0.001, $\eta_{p}^{2}$ =0.052 for treatment; *F*(1, 598) = 27.855, *p* < 0.001, $\eta_{p}^{2}$ =0.045 for visual field], while the main effect of lexicality was non-significant [*F*(1, 598) = 2.091, *p* = 0.149, $\eta_{p}^{2}$ =0.003]. Post-hoc analysis on the two-way interaction effect between treatment and lexicality revealed that responses were significantly more accurate in the nonword LDT compared to the pseudoword LDT for both words and nonwords [*F*(1, 1,198) = 8.781, *p* = 0.003, $\eta_{p}^{2}$ =0.007 for words; *F*(1, 1,198) = 55.781, *p* < 0.001, $\eta_{p}^{2}$ =0.044 for nonwords]. Furthermore, simple main effect analysis on the two-way interaction effect between lexicality and visual field demonstrated a significant difference in accuracy rates between RVF and LVF only for words, but not for nonwords [*F*(1, 599) = 59.271, *p* < 0.001, $\eta_{p}^{2}$ =0.090 for words; *F*(1, 599) = 0.205, *p* = 0.651, $\eta_{p}^{2}$ =0.000 for nonwords].

#### 2.2.2. Signal detection measures (sensitivity and response bias)

**Figure 1:**
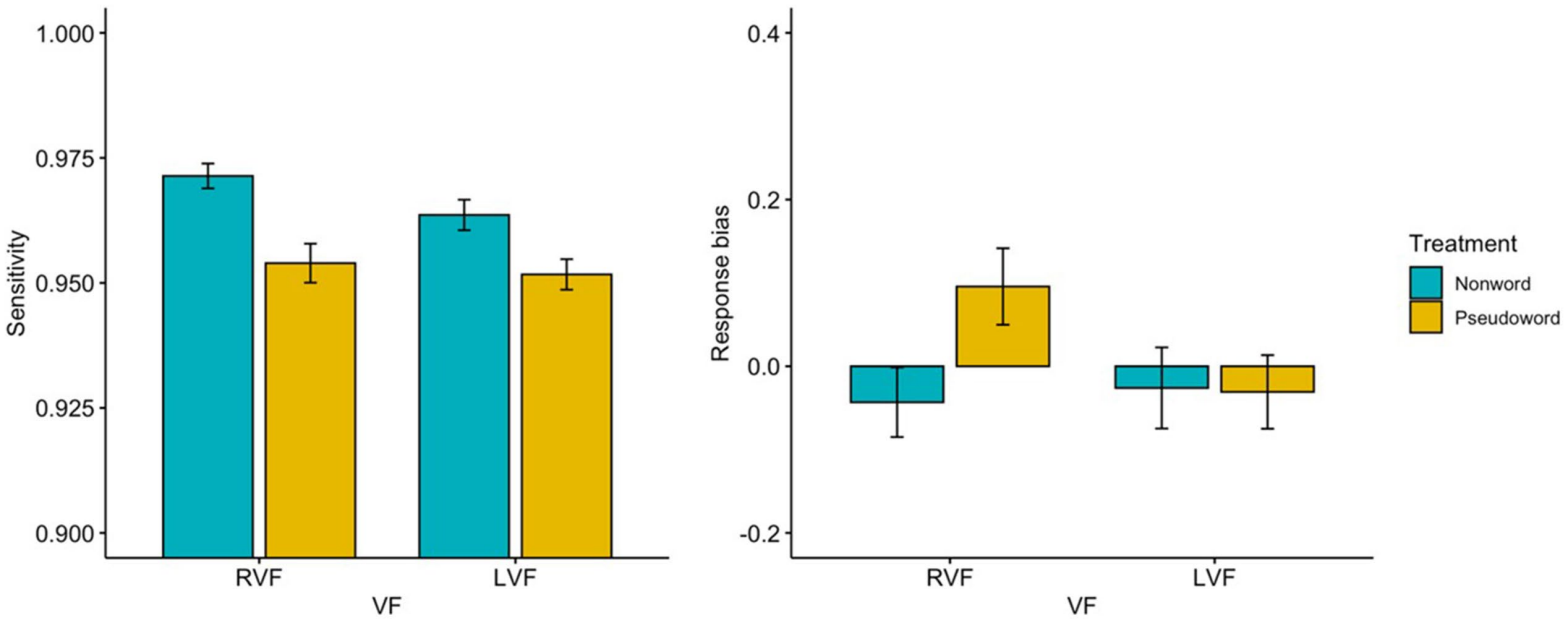
Results of the sensitivity and the response bias at the parafoveal lexical decision (RVF: right visual field, LVF: left visual field) in Experiment 1-1 (nonword) and in Experiment 1-2 (pseudoword). Error bars represent the standard error.

Table 3 and Figure 1 depict the signal detection measures in Experiment 1. The current study employed a mixed-effect ANOVA to investigate the effects of treatment and visual field on sensitivity and response bias in a lexical decision task. Firstly, sensitivity was assessed in a 1-between (treatment: nonword LDT vs. pseudoword LDT) 1-within (visual field: RVF vs. LVF) design. The results indicated no significant interaction effect between treatment and lexicality on sensitivity [*F*(1, 113) = 3.413, *p* = 0.067, $\eta_{p}^{2}$ =0.029]. However, the main effects of treatment [*F*(1, 113) = 12.487, *p* = 0.001, $\eta_{p}^{2}$ =0.100] and visual field [*F*(1, 113) = 24.293, *p* < 0.001, $\eta_{p}^{2}$ =0.177] were significant. Specifically, the nonword LDT yielded higher sensitivity than the pseudoword LDT, and RVF presentation led to higher sensitivity than LVF presentation.

**Table 3:** Results of the sensitivity and the response bias at the parafoveal lexical decision (RVF; right visual field, LVF; left visual field) in Experiment 1–1 (nonword) and Experiment 1–2 (pseudoword).

	Experiment 1–1 (nonword)		Experiment 1–2 (pseudoword)	
	Visual field		Visual field	
	RVF	LVF	RVF	LVF
Sensitivity	0.971 (0.019)	0.962 (0.023)	0.954 (0.029)	0.950 (0.024)
Response bias	−0.043 (0.326)	−0.061 (0.334)	0.096 (0.337)	0.008 (0.368)

The values within brackets denote standard deviation.

Secondly, response bias was evaluated in a 1-between (treatment: nonword LDT vs. pseudoword LDT) 1-within (visual field: RVF vs. LVF) mixed-effect ANOVA. The results showed no significant interaction effect between treatment and visual field on response bias [*F*(1, 113) = 1.091, *p* = 0.298, $\eta_{p}^{2}$ =0.010]. The main effects of treatment [*F*(1, 113) = 3.676, *p* = 0.058, $\eta_{p}^{2}$ =0.032] and visual field [*F*(1, 113) = 2.500, *p* = 0.117, $\eta_{p}^{2}$ =0.022 for visual field] were also not significant. However, one sample t-tests for testifying the statistical significance of the response bias effect revealed a significant response bias at the RVF but not at the LVF in the pseudoword LDT [*t*(53) = 2.085, *p* = 0.042 for the RVF; *t*(53) = 0.153, *p* = 0.879 for the LVF], while the response biases at both unilateral visual fields in the nonword LDT were not significant [*t*(60) = −1.035, *p* = 0.305 for the RVF; *t*(60) = −1.429, *p* = 0.158 for the LVF]^1^.

### 2.3. Discussion

#### 2.3.1. Summary of results in Experiment 1

Experiment 1 aimed to investigate the criteria for lexical decision in the parafoveal visual field by manipulating the orthographical legality of nonwords in two types of LDTs. Behavioral responses, including response times and accuracy, as well as signal detection measures, namely sensitivity and response bias, were measured. The results indicated a significant advantage for the RVF over the LVF in all the behavioral responses and sensitivity measures in both types of parafoveal LDTs. These findings support previous research suggesting left-dominance in language processing and the advantages of RVF/LH word recognition for lexical access. Notably, a significant response bias toward “yes (word)” was observed in the RVF/LH during the pseudoword LDT compared to the nonword LDT. This finding highlights the importance of considering response bias when interpreting LDT results.

#### 2.3.2. Distinct strategies between LH and RH in lexical decision-making

Our findings reveal that the response bias differentially affects the unilateral visual fields, suggesting distinct hemispheric strategies in lexical decision-making. Specifically, our results may demonstrate the dominance of LH in lexical processing, as LH possesses crucial information on orthographic legality for discriminating words from pseudowords. This information appears to be advantageous for lexical decision-making in the pseudoword LDT, resulting in superior performance at the RVF/LH compared to the LVF/RH. Conversely, we did not observe such a response bias in the nonword LDT, implying that discrimination between words and nonwords may not primarily require the use of orthographic legality information due to the distinct differences in visual attributes other than the orthographic legality. Thus, LH seems to employ a different strategy for lexical decision-making, which does not rely on orthographic legality, resulting in superior performance at RVF/LH compared to LVF/RH in the nonword LDT.

Furthermore, our results reveal that RH did not exhibit any response bias at the parafoveal visual fields in both types of LDTs. This suggests that RH may utilize a different processing strategy compared to LH, at least during the parafoveal pseudoword LDT, where no response bias was observed at LVF/RH. We hypothesize that RH may be more lateralized toward processing visual-perceptual attributes and/or visual familiarity of stimuli, as previously suggested in the literature (Hellige and Webster, 1979; Warrington and James, 1986). Moreover, we propose that lexical decision-making in LVF/RH occurs at an early stage of visual processing, primarily utilizing visual-perceptual or familiarity information of stimuli. This may lead to inaccurate and slower lexical decision-making, as well as reduced sensitivity in discriminating between words and nonwords, due to less proficient lexical processing in RH. Our findings suggest that both hemispheres can adopt independent processing strategies for lexical decision-making between words and nonwords, raising the question of whether foveal lexical decisions follow a particular hemispheric strategy.

In Experiment 2, we aimed to explore how the asymmetric processing strategies between the two hemispheres are coordinated during lexical decision-making. We hypothesized that foveal lexical decision-making is primarily dependent on LH, given its propensity to process crucial lexical information required for efficient word recognition. Accordingly, we expected to observe a response bias toward “yes (word)” during the foveal pseudoword LDT in LH, while no such response bias was expected during the foveal nonword LDT, where orthographic legality information is not utilized.

## 3. Experiment 2

The objective of Experiment 2 was to investigate whether foveal lexical decision-making is governed by the processing strategy of LH or RH. To this end, we administered two types of lexical decision tasks, namely the pseudoword and nonword LDTs. We posited that if foveal lexical decision-making follows the processing strategy of LH, it would exhibit a response bias toward “yes (word)” during the foveal pseudoword LDT, in contrast to the foveal nonword LDT. Conversely, if foveal lexical decision-making follows the processing strategy of RH, no response bias would be expected in either of the LDTs.

### 3.1. Methods

#### 3.1.1. Participants

Experiment 2 comprised two sub-experiments (Experiment 2–1 and Experiment 2–2). In Experiment 2–1, a total of 36 participants participated, of whom four failed to comply with the experimental procedures and were excluded from the final data analysis. Thus, data from 32 participants (male: 7, female: 25) were analyzed, with an average age of 24.41 years (SD: 2.91). All participants were strongly right-handed, as ascertained by the Edinburgh handedness inventory (M: 8.28, SD: 1.73). In Experiment 2–2, 32 participants participated, of whom four were excluded from the final data analysis due to noncompliance. Thus, data from 28 participants (male: 11, female: 17) were analyzed, with a mean age of 24.93 years (SD: 2.54). Participants in Experiment 2–2 also showed right-handedness (M: 7.48, SD: 2.15), as assessed by the Edinburgh Inventory. Notably, there were no overlapping participants between Experiment 2–1 and Experiment 2–2.

All participants included in the present study reported having normal or corrected-to-normal vision in both eyes, and no history of neurological impairments. The study was conducted in accordance with the ethical principles outlined in the 1964 Declaration of Helsinki, and all participants provided informed consent prior to their involvement. A nominal monetary compensation was provided to participants for their participation. Exclusion criteria were as follows: (1) history of neurological impairment due to brain damage or stroke, (2) sensory organ impairments, (3) diagnosis of mental illness, and (4) history of substance abuse or addiction.

#### 3.1.2. Experimental task and procedure

Experiments 2–1 and 2–2 aimed to investigate the hemispheric strategies involved in performing foveal lexical decision tasks using different types of nonwords. Experiment 2–1 employed orthographically illegal letter sequences as nonwords, whereas Experiment 2–2 utilized orthographically legal letter sequences as pseudowords. Both experiments employed identical procedures, beginning with a fixation point presented at the center of the screen for 500 ms, followed by arbitrary letter strings displayed for 180 ms at the center of the screen. Following the disappearance of the letter strings, participants were required to determine whether the letter strings were a word or not within 2,000 ms. The letter strings were presented within 1.5° vertical and 2° horizontal visual angles, corresponding to foveal vision.

#### 3.1.3. Apparatus

Experiment 2 utilized the same apparatus as Experiment 1.

#### 3.1.4. Stimuli

In Experiment 2, the same stimuli as in Experiment 1 were utilized. Specifically, Experiment 2–1 involved a set of 300 Korean visual words and 300 pseudowords, while Experiment 2–2 included 300 Korean visual words and 300 nonwords.

#### 3.1.5. Signal detection theory

The indicators of sensitivity and response bias from signal detection theory that were utilized in Experiment 1 were also employed in Experiment 2. The formulas for calculating these indicators were previously described in formulas (1) and (2).

#### 3.1.6. Statistical analyses

For the analyses in Experiment 2, we conducted mixed ANOVAs on behavioral responses (RTs and Acc) with a 1-between (treatment: nonword LDT vs. pseudoword LDT) and 1-within (lexicality: word vs. nonword) design, employing the same factors as in Experiment 1. Furthermore, we analyzed the signal detection measures (sensitivity and response bias) using a 1-between (treatment: nonword LDT vs. pseudoword LDT) design, with the same treatment factor as in Experiment 1.

### 3.2. Results

#### 3.2.1. Behavioral responses (response times and accuracy)

The behavioral response results in Experiment 2 are depicted in Table 4. The response times of participants were subjected to 1-between (treatment: nonword LDT vs. pseudoword LDT) and 1-within (lexicality: word vs. nonword) mixed ANOVAs in Experiment 2. The results revealed a significant two-way interaction effect between treatment and lexicality [*F*(1, 598) = 113.991, *p* < 0.001, $\eta_{p}^{2}$ =0.160], as well as significant main effects of treatment and lexicality [*F*(1, 598) = 300.843, *p* < 0.001, $\eta_{p}^{2}$ =0.335 for treatment; *F*(1, 598) = 265.423, *p* < 0.001, $\eta_{p}^{2}$ =0.307 for lexicality]. Further simple main effect analysis on the significant two-way interaction effect between treatment and lexicality showed that participants responded significantly faster in nonword LDT than in pseudoword LDT for both words and nonwords [*F*(1, 598) = 29.626, *p* < 0.001, $\eta_{p}^{2}$ =0.047 for words; *F*(1, 598) = 324.047, *p* < 0.001, $\eta_{p}^{2}$ =0.351 for nonwords]. The main effect of treatment indicated faster response times in nonword LDT than in pseudoword LDT, while the main effect of lexicality indicated faster response times for words than for nonwords.

**Table 4:** Results of the response times and the accuracy at the foveal presentation (CVF; central visual field) in Experiment 2–1 (nonword) and Experiment 2–2 (pseudoword).

		Experiment 2–1 (nonword)	Experiment 2–2 (pseudoword)
		Visual field	Visual field
		CVF	CVF
Word	RTs	507 (35)	525 (46)
	ACC	0.932 (0.076)	0.924 (0.091)
Nonword	RTs	522 (37)	597 (62)
	ACC	0.922 (0.084)	0.858 (0.145)

The values within brackets denote standard deviation.

Subsequently, we conducted 1-between (treatment: nonword LDT vs. pseudoword LDT) 1-within (lexicality: word vs. nonword) mixed ANOVAs on the accuracy of the responses. Results indicated a significant two-way interaction effect between treatment and lexicality [*F*(1, 598) = 22.886, *p* < 0.001, $\eta_{p}^{2}$ =0.037], as well as significant main effects of treatment and lexicality [*F*(1, 598) = 37.034, *p* < 0.001, $\eta_{p}^{2}$ =0.058 for treatment; *F*(1, 598) = 40.922, *p* < 0.001, $\eta_{p}^{2}$ =0.064 for lexicality]. Simple main effect analysis on the significant two-way interaction effect revealed no significant difference in accuracy for words between nonword and pseudoword LDTs [*F*(1, 598) = 1.259, *p* = 0.262, $\eta_{p}^{2}$ =0.002]. However, it did show significantly higher accuracy for nonwords in nonword LDT than in pseudoword LDT. The main effect of treatment indicated greater accuracy in the nonword LDT than in the pseudoword LDT. Additionally, the main effect of lexicality showed higher accuracy for words than for nonwords.

#### 3.2.2. Signal detection measures (sensitivity and response bias)

**Figure 2:**
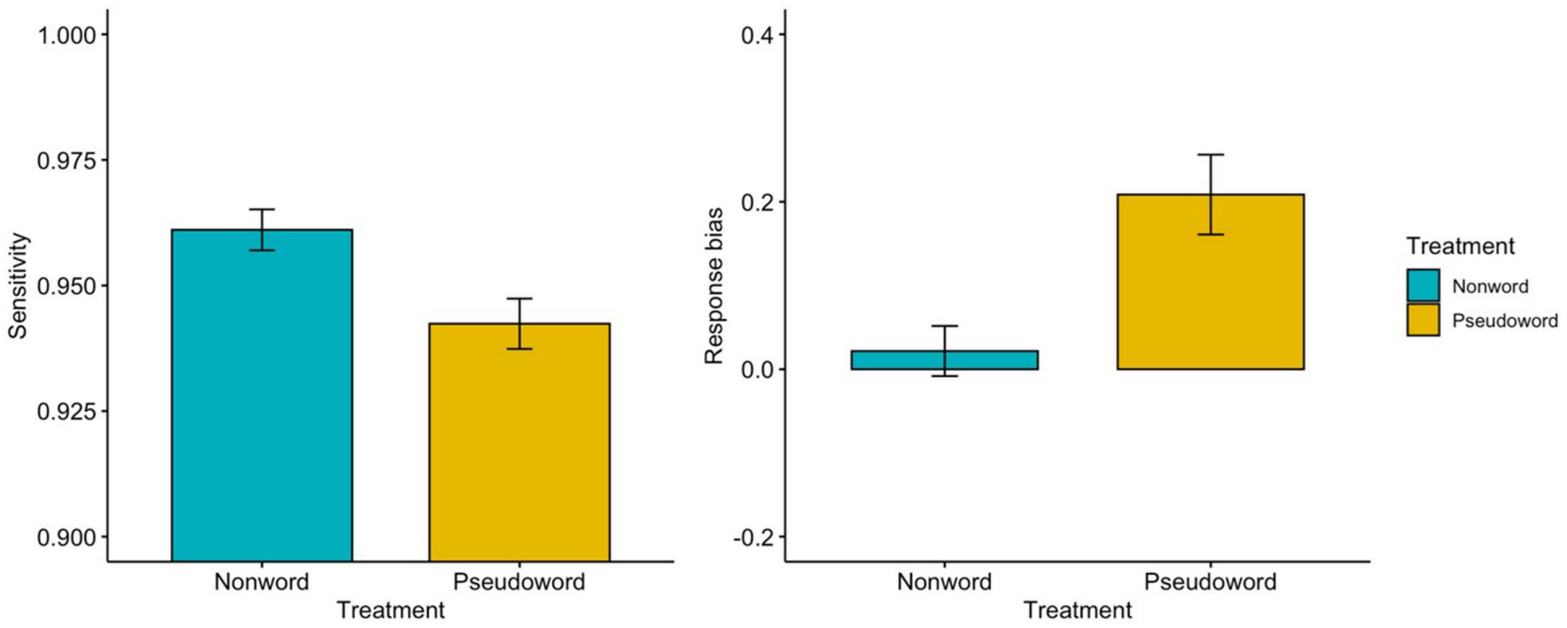
Results of the sensitivity and the response bias at the parafoveal lexical decision (RVF: right visual field, LVF: left visual field) in Experiment 2-1 (nonword) and in Experiment 2-2 (pseudoword). Error bars represent the standard error.

Signal detection measures in Experiment 2 are presented in Table 5 and Figure 2. Firstly, one-way ANOVAs with 1-between factor (treatment: nonword LDT vs. pseudoword LDT) were conducted on the sensitivity in Experiment 2. The analysis yielded a significant main effect of treatment [*F*(1, 58) = 8.556, *p* = 0.005, $\eta_{p}^{2}$ =0.129], indicating that participants had greater sensitivity in the nonword LDT than in the pseudoword LDT. Secondly, one-way ANOVAs with 1-between factor (treatment: nonword LDT vs. pseudoword LDT) were conducted on the response bias. The analysis revealed a significant main effect of treatment [*F*(1, 58) = 11.602, *p* = 0.001, $\eta_{p}^{2}$ =0.167], indicating that participants had a higher response bias toward “yes (word)” in the pseudoword LDT than in the nonword LDT. In addition, one sample t-test of response bias in the foveal LDTs only found a significant effect in the foveal pseudoword LDT in comparison to the foveal nonword LDT [*t*(31) = 0.725, *p* = 0.474 for the foveal nonword LDT; *t*(27) = 4.374, *p* < 0.001 for the foveal pseudoword LDT].

**Table 5:** Results of the sensitivity and the response bias at the foveal lexical decision (CVF; central visual field) in Experiment 2–1 (nonword) and Experiment 2–2 (pseudoword).

	Experiment 2–1 (nonword)	Experiment 2–2 (pseudoword)
	Visual field	Visual field
	CVF	CVF
Sensitivity	0.961 (0.023)	0.942 (0.026)
Response bias	0.022 (0.169)	0.209 (0.252)

The values within brackets denote standard deviation.

### 3.3. Discussion

#### 3.3.1. Summary of results in experiment 2

In Experiment 2, our aim was to investigate whether foveal lexical decision engages the strategy of LH or RH, by presenting two types of lexical decision tasks: the pseudoword and nonword LDTs. Firstly, we observed a significant two-way interaction effect between treatment and lexicality in both response times and accuracy, indicating slower and less accurate responses to pseudowords than to nonwords. This finding supports the reliability of the foveal results in our study. Secondly, we found significantly lower sensitivity and a response bias toward “yes (word)” in the foveal pseudoword LDT compared to the foveal nonword LDT. These results suggest that foveal lexical decision relies on the orthographical legality strategy, which is predominantly employed by LH.

#### 3.3.2. Potential involvement of RH in lexical decision as a subordinate hemisphere

These findings implicated the potential involvement of RH in foveal lexical decision. The results indicated a higher sensitivity in the nonword LDT and no significant response bias although a similar sensitivity in the nonword LDT and the pseudoword LDT was expected due to predominance of LH in foveal word processing. The higher sensitivity in the nonword LDT suggests the participation of RH in discriminating between word and nonword in contrast between word and pseudoword, indicating a meaningful use of supportive processing in RH for lexical decision between word and nonword. These findings suggest that the involvement of RH in foveal lexical decision is task-dependent and determined by the demands of the nondominant hemisphere in foveal word processing. These results align with previous research reporting asymmetrical excitatory transfer from RH to LH in language processing (Nowicka and Tacikowski, 2011). The present study observed faster response times and higher accuracy in the nonword LDT than the pseudoword LDT, suggesting that the asymmetrical excitatory transfer from RH may facilitate rather than inhibit the work of the bilateral hemisphere (BH), supporting the cooperation model in interhemispheric processing for lexical decision instead of the parallel and inhibition processing models.

The results of Experiment 2 provide support for the hypothesis that the foveal presentation of both pseudoword and nonword LDTs recruits LH’s orthographical legality strategy for lexical decision. However, our findings suggest that the involvement of RH in foveal word processing is dependent on task demands. Discriminating between words and pseudowords requires access to lexical information that is less available in RH than LH. As such, RH may not function optimally when processing pseudoword LDTs, despite its potential usefulness in nonword LDTs, given their reliance on visual-perceptual attributes or familiarity (Hellige and Webster, 1979; Warrington and James, 1986). Thus, the different task demands of LDTs appear to differentiate the hemisphere involvement in lexical decision.

Experiment 2 provides evidence that LH is dominant in storing and utilizing lexical information for efficient foveal lexical decision-making. Moreover, the results suggest that bilateral hemisphere (BH) interacts to determine which hemisphere is better suited for the task at hand. Once identified, BH asymmetrically may engage both hemispheres’ specializations, differentiating between dominant and nondominant hemispheres, to facilitate lexical decision-making.

In Experiment 2, we investigated the interhemispheric processing of foveal words by manipulating task demands through pseudoword and nonword LDTs. The results suggest that left and right hemisphere strategically selects the dominant hemisphere to process visual words based on the usefulness of lexical information. However, this does not imply that the other hemisphere is completely uninvolved in the processing. Rather, left and right hemisphere appears to coordinate the asymmetrical magnitude of work due to their different specialties in lexical decision. The asymmetric coordination between left and right hemisphere supports a common goal, namely, foveal lexical decision. Experiment 2 revealed the different involvement of the two hemispheres in foveal processing, depending on the type of nonword in the LDT. This suggests that the dynamics of interhemispheric interactions determine which hemisphere’s processing strategy is employed and how they perform with their employed specialty. These findings contribute to a better understanding of the interhemispheric processing of foveal words and highlight the importance of considering task demands in investigating hemispheric contributions to language processing.

## 4. General discussion

### 4.1. Summary of the findings in parafoveal and foveal lexical decision experiments

This study aimed to investigate the interhemispheric interaction of left and right hemisphere in lexical decision-making, utilizing parafoveal and foveal presentations. By manipulating the orthographical legality of letter strings, two types of nonwords were presented in both foveal and parafoveal lexical decision tasks: orthographically illegal nonwords (nonwords) and orthographically legal nonwords (pseudowords). In Experiment 1, the visual half-field presentation paradigm was employed to explore the different strategies between the two hemispheres. Results indicated that LH utilized orthographical legality information for lexical decision-making in the parafoveal pseudoword LDT, whereas RH did not show any response bias toward a particular response in both parafoveal pseudoword and nonword LDTs. Experiment 2 aimed to investigate how the two hemispheres interact with each other in foveal lexical decision-making, by observing whether the foveal lexical decision follows the strategy of LH or RH using foveal pseudoword and nonword LDTs. Results showed a significant response bias toward “word” in the pseudoword LDT, indicating that left and right hemisphere employed the left-centered orthographical legality strategy for lexical decision-making. Furthermore, RH was found to play a supportive role for lexical decision-making in the nonword LDT, indicating that its participation may be manipulated by task demand. These findings suggest that left and right hemisphere strategically distinguishes the dominant hemisphere and the nondominant hemisphere by determining which hemisphere should be mainly employed in lexical decision-making.

### 4.2. Why task demand matters in lexical decision and its relation to hemispheric processing

In the field of visual word recognition, there are various models that explain how humans process words and nonwords differently. Grainger and Jacobs (1996) and Coltheart et al. (2001) propose that the degree of word-likeness of nonword stimuli is an important factor in the decision-making process. Specifically, when presented with a pseudoword that has a high degree of word-likeness, the reliability of general lexical activation decreases, and individuals rely more on the activation of specific sublexical representations such as letters. This prompts a shift toward local activation of specific letter combinations, rather than relying on global lexical activation. Additionally, Ratcliff et al. (2004) proposed that the word-likeness of pseudowords would result in a more conservative response threshold, requiring more lexical evidence to make a lexical decision. This is due to the fact that the activation level of the specific sublexical representations is weaker for pseudowords than for real words, making it more difficult to determine whether a presented stimulus is a real word or not. These models suggest that the degree of word-likeness in pseudoword stimuli affects the decision-making process during visual word recognition. Furthermore, the models may suggest that the differential interhemispheric interactions are dependent on the nonword type and task difficulty. The degree of word-likeness in pseudowords determines how lexical decisions are made based on the degree of lexical activation by focusing on local attributes and response threshold, thus expecting differential involvement of the two hemispheres for lexical decision. This is because the two hemispheres are expected to possess asymmetric strategies in lexical processing for visual words.

The current investigation provides new insights into the distinct cognitive strategies employed by the left and right hemispheres in the context of lexical decision-making, with a particular emphasis on their use of orthographic legality information. Furthermore, it illuminates the coordination of both hemispheres during foveal lexical decision-making. Our data demonstrate a bias toward “word” response during foveal presentation, which is congruent with the influence of left hemisphere processing. The findings suggest that the left hemisphere’s superior performance in lexical processing may be ascribed to its preferential utilization of its strategy during foveal lexical decision-making. Additionally, due to the greater availability of relevant lexical information in the left hemisphere compared to the right, the involvement of the right hemisphere in foveal word processing may necessitate more resources than relying on the left hemisphere’s strategy. In this regard, Kim et al. (2023) offer electrophysiological evidence supporting superior facilitation from the right to the left hemisphere during more familiar word recognition at foveal vision, which may lead to left-lateralization for efficient foveal word processing. Furthermore, parafoveal studies have shown that word processing operates cooperatively between the two hemispheres to achieve efficient processing by modulating word familiarity (Kim et al., 2022) and semantic information of words (Perrone-Bertolotti et al., 2013) with the significant RVFA. These studies suggest that interhemispheric coordination with a left hemisphere focus may be present.

This investigation reveals the potential contribution of RH in foveal lexical decision tasks, specifically in distinguishing between word and nonword stimuli. Intriguingly, the involvement of RH appears to enhance left-centered processing during foveal nonword lexical decision tasks, as compared to pseudoword lexical decision tasks. This finding suggests a collaborative interplay between the hemispheres, wherein the nondominant hemisphere supports the dominant hemisphere in executing the task. The results of this study provide evidence that foveal word processing primarily relies on LH as the dominant hemisphere for language processing, while RH may be selectively recruited as the nondominant hemisphere for lexical decision tasks. This asymmetrical coordination between the hemispheres aligns with the direction of information transfer assumed in language processing, from the nondominant hemisphere to the dominant hemisphere (Nowicka and Tacikowski, 2011).

The present investigation elucidates the model of asymmetrical cooperation in interhemispheric processing during foveal lexical decision, wherein left-lateralized asymmetric collaboration from RH to LH is highlighted. This asymmetrical interplay implies a potential regulation of interhemispheric interaction, attributable to the unidirectional pattern of processing observed across the hemispheres during foveal lexical decision tasks. The left-lateralized processing indicates the integration of LH processing, subsequent to input transmission to RH, underscoring the superior language processing capabilities of LH. The findings of this study provide compelling evidence for the asymmetric involvement of interhemispheric cooperation, with a predominant contribution from the dominant hemisphere and a relatively smaller role from the nondominant hemisphere, potentially underpinning the interhemispheric cooperation mechanism in lexical decision tasks.

### 4.3. Implications of the current study in terms of hemispheric processing in lexical decision

In this study, we have identified several key implications related to the lexical decision task. Firstly, our results demonstrate the strategic use of orthographical legality by LH during the task. Specifically, we found that participants exhibited a response bias toward the word category in the RVF during the pseudoword LDT, but not during the nonword LDT. Secondly, our findings suggest that this strategy based on orthographical legality is also recruited during foveal lexical decision. We observed a significant response bias toward the word category during the foveal pseudoword LDT, as compared to the foveal nonword LDT. Finally, our results provide evidence for potential interhemispheric interaction during foveal lexical decision, with support from RH for the nonword LDT relative to the pseudoword LDT. These findings suggest that RH participation may be modulated by task demand.

## 5. Conclusion

In conclusion, this study has shed light on the interhemispheric processing involved in lexical decision making. The results highlight the importance of left-lateralized processing in the dominant hemisphere (LH) for effective language processing during the lexical decision. Furthermore, the study revealed that the recruitment of a strategy based on orthographical legality information is crucial in the foveal lexical decision. The investigation also suggests that the nondominant hemisphere (RH) may participate in lexical decision-making when task demands require it, and the interhemispheric cooperation between the two hemispheres may occur asymmetrically, with a larger contribution from the dominant hemisphere and a lesser work from the nondominant hemisphere. The current findings provide a foundation for future research exploring the intricate mechanisms involved in interhemispheric processing during the lexical decision.

## Data availability statement

The original contributions presented in the study are included in the article/Supplementary material, further inquiries can be directed to the corresponding author.

## Ethics statement

The studies involving human participants were reviewed and approved by Korea University Institutional Review Board. The patients/participants provided their written informed consent to participate in this study.

## Author contributions

SK: conceptualization, methodology, investigation, formal analysis, project administration, visualization, and writing— original draft, review, and editing. KN: conceptualization and supervision. All authors contributed to the article and approved the submitted version.

## Funding

This research was supported by the MSIT (Ministry of Science and ICT), South Korea, under the ITRC (Information Technology Research Center) support program (IITP-2022-2017-0-01630) supervised by the IITP (Institute for Information and communications Technology Promotion) and by the Ministry of Education of the Republic of Korea and the National Research Foundation of Korea (NRF-2022S1A5B5A16049384).

## Conflict of interest

The authors declare that the research was conducted in the absence of any commercial or financial relationships that could be construed as a potential conflict of interest.

## Publisher’s note

All claims expressed in this article are solely those of the authors and do not necessarily represent those of their affiliated organizations, or those of the publisher, the editors and the reviewers. Any product that may be evaluated in this article, or claim that may be made by its manufacturer, is not guaranteed or endorsed by the publisher.

## Footnotes

^1^In the field of perception and cognition research, understanding and interpreting response bias is critical as it provides insight into a participant’s tendency to respond in a particular way, regardless of their sensitivity to stimuli. Unlike sensitivity, the interpretation of response bias requires a one-sample t-test to determine the significance of the effect, since the absolute size of the effect is a better reflection of the measure’s meaning. Specifically, to interpret response bias as a significant effect, it is necessary to determine whether there is a significant difference between the response bias value and zero. This is important because if there is no significant difference, it means that there is no practical bias even if the response bias value is measured. Therefore, it is necessary to conduct one-sample t-tests in addition to ANOVA analyses to verify response bias within a specific condition. This ensures that the response bias measure accurately represents the participant’s responses and can be used to draw valid conclusions about their perceptions and cognition.
